# Unique inflammatory signature in haemophilic arthropathy: miRNA changes due to interaction between blood and fibroblast‐like synoviocytes

**DOI:** 10.1111/jcmm.16068

**Published:** 2020-11-07

**Authors:** Sandra Mignot, Nicolas Cagnard, Benoit Albaud, Cécile Bally, Justine Siavellis, Olivier Hermine, Laurent Frenzel

**Affiliations:** ^1^ Laboratory of Cellular and Molecular Mechanisms of Hematological Disorders and Therapeutical Implications Paris Descartes – Sorbonne Paris Cité University, Labex GR‐Ex, Imagine Institute, Inserm U1163 Paris France; ^2^ Bioinformatics Platform – Institut IMAGINE Paris France; ^3^ Genomic Platform – Institut CURIE Paris France; ^4^ Hematology unit care – hemophilia Center – Necker Hospital Paris France; ^5^ Faculté de médecine Paris‐Descartes Paris France

**Keywords:** cytokines, FLS, Haemophilia, haemophilic arthropathy, miRNA

## Abstract

In haemophilia, the recurrence of hemarthrosis leads to irreversible arthropathy termed haemophilic arthropathy (HA). However, HA is a unique form of arthropathy in which resident cells, such as fibroblast‐like synoviocytes (FLS), come into direct contact with blood. Therefore, we hypothesized that FLS in HA could have a unique inflammatory signature as a consequence of their contact with blood. We demonstrated with ELISA and ELISPOT analyses that HA‐FLS expressed a unique profile of cytokine secretion, which differed from that of non‐HA‐FLS, mainly consisting of cytokines involved in innate immunity. We showed that unstable cytokine mRNAs were involved in this process, especially through miRNA complexes as confirmed by DICER silencing. A miRNOME analysis revealed that 30 miRNAs were expressed differently between HA and non‐HA‐FLS, with most miRNAs involved in inflammatory control pathways or described in certain inflammatory diseases, such as rheumatoid arthritis or lupus. Analysis of transcriptomic networks, impacted by these miRNAs, revealed that protein processes and inflammatory pathways were particularly targeted in LPS‐induced FLS, and in particular vascularization and osteoarticular modulation pathways in steady‐state FLS. Our study demonstrates that the presence of blood in contact with FLS may induce durable miRNA changes that likely participate in HA pathophysiology.

## 1 INTRODUCTION

Haemophilia is an X‐linked bleeding disease caused by a partial or complete deficiency of coagulation factor VIII or IX.^1^ Bleeding mainly occurs in large joints, such as the knee, elbow or ankle, as a result of inadequate thrombin generation due to the lack of a clotting factor.^2^

Recurring hemarthrosis leads to an irreversible arthropathy, termed haemophilic arthropathy (HA), characterized by a progressively complete destruction of the joint with permanent pain, musculoskeletal disability and poor quality of life. Although prophylactic treatment with a clotting factor *versus* episodic infusion has demonstrated superiority in preventing joint disease,^3^ HA remains a significant comorbidity of the haemophilic patient.

Indeed, the pathophysiology of blood‐induced joint disease remains unclear. Some data suggest that the pathobiology of HA may be similar to the pathogenicity of rheumatoid arthritis (RA), given that chronic proliferative synovitis and cartilage destruction are present in both.^4^ Moreover, metalloprotease enzymes, inflammatory cells and inflammatory cytokines, such as interleukin‐1 (IL‐1) or tumour necrosis factor alpha (TNF‐α), have been found in the synovium of HA as would be expected in RA.^5^ One of the main difference between the two diseases is the origin of inflammation: autoimmunity in RA and blood injury in HA. Synovial iron deposition as a result of blood in the joint is considered the main component in triggering and sustaining the inflammatory response and cell proliferation within the synovial membrane.^6^ Thus, synovial macrophages from blood injury promote the inflammatory pathway of hemarthrosis and HA. However, these elements alone cannot explain the entire pathophysiology of HA. Indeed, in hemochromatosis, another arthropathy characterized by synovial iron deposition, there is no evidence of significant synovial hyperplasia, although inflammatory cells and articular destruction have been observed.^7^ If we focus on the main difference between these two forms of arthritis, the only element found purely in HA is the presence of blood in the joint in direct contact with the synovial membrane, and, particularly, with the unique resident cells in joints, the fibroblast‐like synoviocytes (FLS).

The exact role of FLS in HA pathophysiology is still unknown, but assumed to be central. As widely demonstrated in RA, FLS straddles the boundary between innate and adaptive immunity, given that they synthesize inflammatory cytokines while cooperating with lymphocytes and macrophages.^8^, ^9^ Owing to their high proliferation rate in HA, these cells are directly involved in synovial proliferation and perpetuate inflammation, thereby contributing to cartilage damage. Due to this proliferative activity, the apoptotic pathway is disrupted to the advantage of anti‐apoptotic activity. C‐MYC and Mdm2, two of the main pro‐mitotic factors, are overexpressed in the FLS of HA.^10^, ^11^ The use of an agonistic anti‐human Fas monoclonal antibody can induce FLS apoptosis in HA and reduce inflammatory cytokine production, thereby abolishing synovial hypertrophy.^12^

We hypothesized that, in haemophilia, chronic interaction between blood and FLS in the joint leads to a unique cytokine signature due to acquired and lasted changes, explaining its inflammatory and proliferative characters. First, from the synovium of patients who underwent orthopaedic surgery, we isolated pure HA‐FLS *versus* non‐HA‐FLS. Following stimulation, the expression pattern of cytokines from HA‐FLS completely differed from that from non‐HA‐FLS, mainly reflected by messenger RNA (mRNA) instability and micro‐RNA (miRNA) interaction. A miRNA microarray assay showed HA as possessing a unique signature closer to the miRNA of patients with chronic inflammatory diseases, such as RA, than other forms of arthritis.

Our data demonstrated that blood could induce durable epigenetic changes in resident cells due to pathological interactions.

## 2 MATERIALS AND METHODS

### 2.1 Reagents

Cell culture media (RPMI 1640, M199), foetal bovine serum, L‐glutamine, penicillin, streptomycin, amphotericin B and trypsin reagent were purchased from Thermo Fisher Scientific. Lipopolysaccharides (LPS) were obtained from Salmonella abortus equi, Type XI collagenase and actinomycin D from Streptomyces sp from Sigma‐Aldrich. The Human Cytokine Array Kit and enzyme immunoassay kit for detecting human IL‐1α, IL‐6 and TNF‐α were obtained from R&D Systems. The iScript Reverse Transcription Supermix for the real‐time quantitative polymerase chain reaction (RT‐qPCR), and SsoFast EvaGreen Supermix, were obtained from Bio‐Rad. The RNeasy Plus Mini Kit and miScript System were obtained from Qiagen (Courtaboeuf, France). Flow cytometry antibodies and corresponding isotypes were obtained from Becton Dickinson (LePontdeClaix, France).

### 2.2 Cell activation

FLS (5.10^5^cells) and THP‐1 (10^7^cells) were stimulated with 1 mL of either medium alone or medium containing LPS (1 µg/mL) for 6 hours. After stimulation, supernatants were harvested and assayed for cytokine content using commercially available ELISA tests.

### 2.3 Flow cytometry

For the expression of the cluster of differentiation CD45, CD55, CD68, CD90 and CD106 in human FLS, HFLS cell lines (RA‐HFLS, HR‐FLS) and THP‐1 cells were measured using a flow cytometry assay.^8^ After culture, the cells were washed twice with phosphate buffered saline (PBS) and then incubated for 20 minutes at room temperature in the dark with a mixture of Krome Orange‐conjugated anti‐CD45, PE‐conjugated anti‐CD55, FITC‐conjugated anti‐CD68, BV421‐conjugated anti‐CD90 and APC‐conjugated anti‐CD106. Then, after two washes with PBS, the fluorescent cells were analysed on a FACScan flow cytometer (Gallios, Beckman Coulter) using Kaluza Analysis (Beckman Coulter).

### 2.4 Protein profiling from supernatant

Protein profiling was performed based on the supernatant of cell activation. Some 300 µL of supernatant were loaded on proteome profiler antibody array membranes (Human XL Cytokine Array Kit, R&D Systems), as suggested by the supplier. These membranes were washed and incubated with biotinylated detection antibody cocktail, streptavidin/horseradish peroxidase and chemiluminescent detection reagents, as suggested by the supplier. Membranes were exposed to a ChemiDoc and analysed using ImageJ Software.

Image analysis was performed with background subtraction and normalization to membrane reference points. Differences in the expression levels of various proteins were calculated pairwise as fold changes, with comparisons made between HA and non‐HA‐FLS.

### 2.5 Stimulation of cells for total extraction

Total RNA was extracted from human FLS or THP‐1 cells incubated for 6 hours with either medium alone or medium containing LPS used the RNeasy Plus Mini Kit according to the manufacturer's instructions. Total RNA isolated from the FLS and THP‐1 cells was reverse transcribed using the iScript Reverse Transcription Supermix for RT‐qPCR, according to the manufacturer's instructions (Bio‐Rad), and amplified.

### 2.6 mRNA decay measurement

The stability of mRNA was assessed by adding 5 µg/mL of actinomycin D to the cell medium following activation with foetal bovine serum to inhibit mRNA transcription. At the indicated time points, it was possible to correlate the relative amount of specific mRNA remaining in each sample with mRNA degradation. Total RNA was extracted 2 hours after treatment with actinomycin D, and endogenous mRNA levels were analysed by RT‐qPCR. Because the mRNA levels for GAPDH and β actin were unchanged after actinomycin D treatment, the GAPDH and β actin gene were employed as a reference, and the ratio of IL‐1α/IL‐6 and GAPDH/β actin in each sample was calculated.

### 2.7 Real‐time quantitative polymerase chain reaction

RT‐qPCR was performed on a total of 20 µL using the SsoFast EvaGreen Supermix (Bio‐Rad) and gene‐specific primers for (i)TNF‐α (5’‐TCC‐TTC‐AGA‐CAC‐CCT‐CAA‐CC‐3’ and 5’‐AGG‐CCC‐CAG‐TTT‐GAA‐TTC‐TT‐3’), (ii)IL‐1α(5’‐CCC‐ACA‐GAC‐CTT‐CCA‐GGA‐GAA‐T‐3’ and 5’‐CGA‐CAC‐CCT‐CGT‐TAT‐CCC‐ATG‐TGT‐CG‐3’), (iii)IL‐6 (5’‐TAC‐CCC‐CAG‐GAG‐AAG‐ATT‐CC‐3’ and 5’‐TTT‐TCT‐GCC‐AGT‐GCC‐TCT‐TT‐3’), (iv)GAPDH (5’‐TTG‐ATT‐TTG‐GAG‐GGA‐TCT‐CG‐3’ and 5’‐GAG‐TCA‐ACG‐GAT‐TTG‐GTC‐GT‐3’) and (v) β ‐actin (5’‐CGT‐ACC‐CAT‐CAC‐GAT‐GCC‐AGT‐GGT‐ACG‐3’ and 5’‐ACG‐TTG‐CTA‐TCC‐AGG‐CTG‐TGC‐3’).

After initial denaturing at 95°C for 3 minutes, the temperatures used were 95°C for 5 seconds, 58°C for 15 seconds and 72°C for 15 seconds. Some 40 cycles were performed using the C1000 Touch Instrument (Bio‐Rad).

RT‐qPCR analyses for miRNAs were conducted using the miScript System, and the primers (Qiagen) and RNA concentrations were determined with a NanoDrop instrument (Thermo Fisher). A 1000 ng RNA sample was employed for the assays. Reverse transcriptase reactions and RT‐qPCR were carried out according to the manufacturer's protocols. An endogenous control was employed for normalization. All reactions were run in triplicate on a C1000 Touch Instrument (Bio‐Rad).

### 2.8 Single interfering RNA transfection

The DICER siRNA used in our study was designed to effectively inhibit DICER activity, being supplied by Qiagen.

Cells were transfected with siRNA using the HiPerFect Transfection Reagent kit (Qiagen). Cells were plated in 24‐well plates (1 × 10^3^ cells/well). All assays were performed 24 hours after transfection.

### 2.9 Statistical analysis

Statistical analysis was performed using Student's *t* test. Values were compared between different groups. A p‐value of <0.05 was considered statistically significant.

### 2.10 Cell culture

Human Fibroblast‐like synoviocytes (FLS) were isolated from the synovial tissues of five different HA patients (3 patients with severe haemophilia A, 1 patient with severe haemophilia B and 1 patient with type 3 of von Willebrand disease) and three non‐HA patients (1 patient with articular disruption of neurological origin, 1 patient with nodular villous synovitis and 1 patient with osteoarthritis) during knee, ankle or hip joint arthroscopic synovectomy, as previously described,^13^ after informed consent had been obtained from patients.

For miRNOMe analysis, in the group HA‐FLS with 3 selected patients, one had a diagnosis of severe haemophilia A (34‐year‐old man, synovial hypertrophy of right ankle), one severe haemophilia B (25‐year‐old man, large synovial hypertrophy of left knee) and one Type 3 of von Willebrand disease (22‐year‐old man, synovial hypertrophy of right ankle) presenting a very close clinical phenotype to that of severe haemophilia A with a haemophilic arthropathy. In the non‐HA‐FLS group with 2 selected patients, one required synovectomy due to articular disruption of neurological origin (18‐year‐old man, left hip) and the other due to nodular villous synovitis (29‐year‐old man, right knee).

Experiments were performed between passages 3 and 9. During that time, cultures comprised a homogeneous population of fibroblastic cells.

Regular Human Fibroblast‐Like Synoviocytes (HR‐FLS) and Rheumatoid Arthritis Fibroblast‐like synovoviocytes (RA‐FLS) (408‐05a, 408RA‐05a, Cell Applications, San Diego, US) were cultured like human FLS. THP‐1 cells (88 081 201, European Collection of Authenticated Cell Cultures, Salisbury, UK) were cultured, as described previously^8^, ^14^ As synovium has 2 types of synoviocytes (macrophage and fibroblasts), THP‐1s were used as control macrophages innate immune cells compared to fibroblasts.

### 2.11 RNA extraction, labelling and miRNA expression profiling analysis

Total RNA was extracted using the supplementary protocol by purifying total RNA containing miRNA from animal cells using the RNeasy Plus Mini Kit. The GeneChip miRNA 4.0 Array (Affymetrix) was applied for miRNA expression profile analysis. This chip contains 30 424 probe sets, including 2578 mature human miRNA sets. After total RNA quality had been inspected, the total RNA of each sample was tailed with poly A and labelled with biotin using the FlashTag Biotin HSR RNA Labeling Kit (Affymetrix), according to the manufacturer's instructions. The hybridization of the bio‐labelled RNA samples was performed according to the manufacturer's instructions for the Affymetrix GeneChip miRNA 4.0 Array with the GeneChip Hybridization Wash and Stain Kit and GeneChip Hybridization Oven 645 (both from Affymetrix). After washing and staining using the GeneChip Fluidics Station 450, the arrays were scanned by means of the GeneChip Scanner 3000 (both from Affymetrix).

Fluorescence data were imported into the Affymetrix Expression Console and R Bioconductor. Gene expression levels were calculated using the Expression Console's RMA DABG algorithm. Comparisons between groups were performed using Student's t test. To estimate the false discovery rate, we filtered the resulting p‐values at 5%. Cluster analysis was performed by hierarchical clustering using the Spearman correlation similarity measure and average linkage algorithm.

### 2.12 Data were available on web Array Express, with the number: E‐MATB‐7442

Model of onset haemophilic arthropathy after repeated contact between plasma and normal HR‐FLS.

Regular Human Fibroblast‐Like Synoviocytes (HR‐FLS) were contacted with plasma repeatedly for 3 weeks. Briefly, HR‐FLS were inoculated in flask, and the next day, a ratio of 50% plasma/50% medium is used for cell culture. Every 3 days, the exposure with fresh plasma with the same ratio is renewed. After 3 weeks, HR‐FLS (5.10^5^ cells) were stimulated with 1 mL of either medium alone or medium containing LPS for 6 hours. Total RNA was extracted following the instructions below. miRNA‐specific primers were obtained from Qiagen.

## 3 RESULTS

### 3.1 HA‐FLS and non‐HA‐FLS are solely composed of a pure homogenous fibroblastic population

As only little data described the use of human FLS from haemophilic patients are available, we first verified that cell cultures of HA and non‐HA‐FLS were composed of pure fibroblastic cell populations. A study of cell surface expression, using flow cytometry, revealed that HA‐FLS (Figure S1A), non‐HA‐FLS (Figure S1B), RA‐FLS (Figure S1C) and HR‐FLS (Figure S1D) exhibited similar cell surface markers (CD45, CD55, CD68, CD90 and CD106), completely differing from the THP‐1 cell line (Figure S1E).^8^

### 3.2 An unique release of inflammatory cytokines in HA‐FLS

**FIGURE 1:**
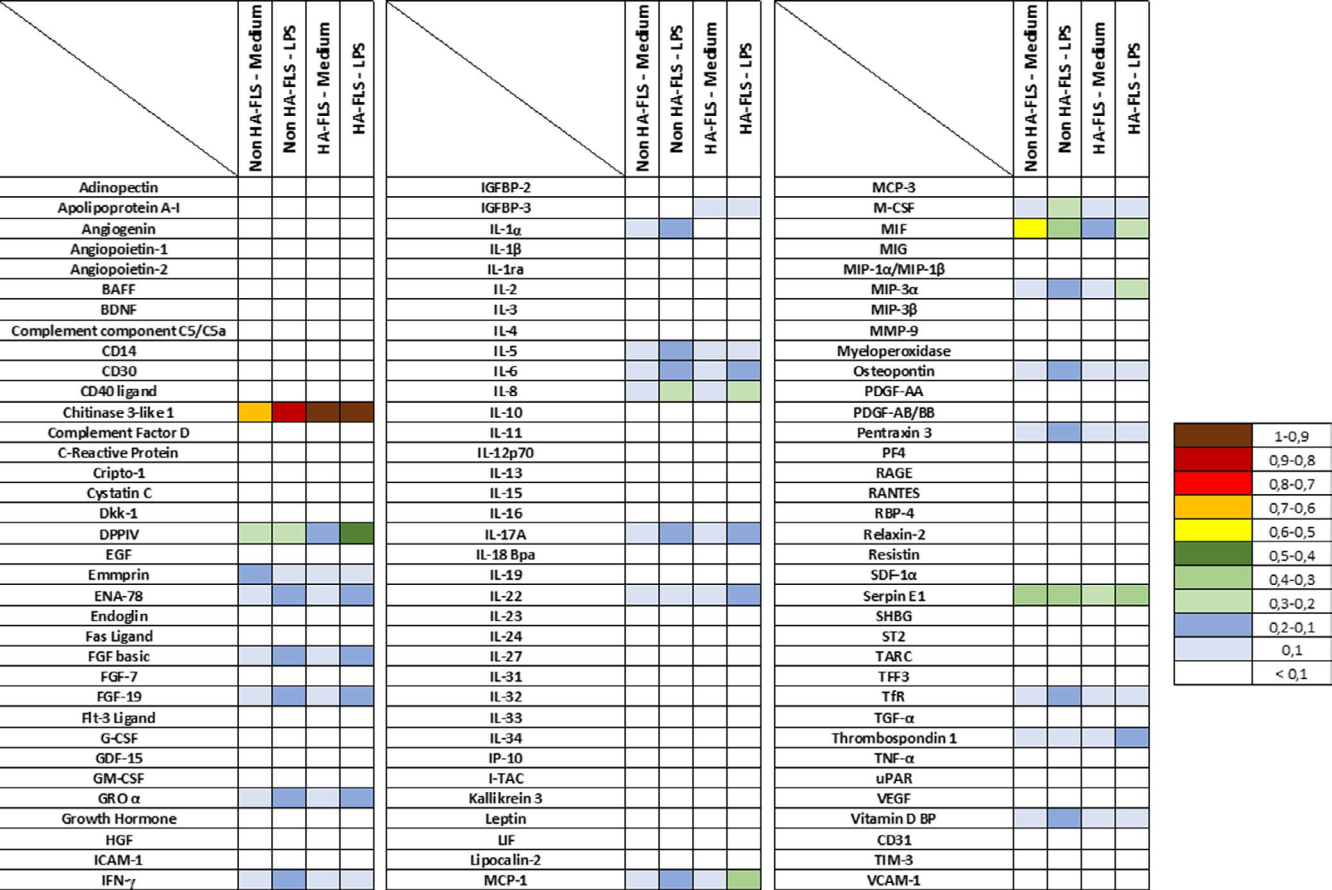
Differences in cytokine expression between HA‐FLS and non‐HA‐FLS. Cells (HA‐FLS and non‐HA‐FLS) are activated with LPS (1 µg/mL) for 6 hours. Cytokines releases were determined by Cytokine Array (The release of 105 cytokines was studied) in culture supernatants. We compared non‐HA‐FLS (3 patients) versus HA‐FLS (5 patients) in two conditions: medium or LPS. The cytokine differences were classified according to their expression: 1 represents the highest expression and < 0.1 means that the cytokine is not released by the cells. HA‐FLS: FLS of haemophilic patients; non‐HA‐FLS: FLS of non‐haemophilic patients; LPS: lipopolysaccharide; BAFF: B‐cell activating factor; BDNF: brain‐derived neurotrophic factor; CD: cluster of differentiation; DPPIV: dipeptidyl peptidase; EGF: epidermal growth factor; ENA: epithelial neutrophil‐activating protein; FGF: fibroblast growth factor; G‐CSF: granulocyte‐colony‐stimulating factor; GDF: growth differentiation factor; GM‐CSF: granulocyte‐macrophage colony‐stimulating factor; GRO: growth‐regulated oncogene; HGF: hepatocyte growth factor; ICAM: intercellular adhesion molecule; IFN: interferon; IGFBP: insulin‐like growth factor‐binding protein; IL: interleukin; IP: interferon‐inducible protein; I‐TAC: interferon‐inducible T‐cell alpha chemoattractant; LIF: leukaemia inhibitory factor; MCP: monocyte chemoattractant protein; M‐CSF: monocyte colony‐stimulating factor; MIF: macrophage migration inhibitory factor; MIG: monokine induced by gamma interferon; MIP: macrophage inflammatory protein; MMP: matrix metallopeptidase; PDGF: platelet‐derived growth factor; PF: platelet factor; RAGE: receptor for advanced glycation end products; RANTES: regulated on activation, normal T cell expressed and secreted; RBP: retinol‐binding protein; SDF: stromal cell‐derived factor; SHBG: sex hormone‐binding globulin; TARC: thymus and activation regulated chemokine; TFF: trefoil factor; TGF: transforming growth factor; TNF‐α: tumour necrosis factor; uPAR: urokinase receptor; VEGF: vascular endothelial growth factor; TIM; T‐cell immunoglobulin and mucin‐domain containing; VCAM: vascular cell adhesion molecule

To examine the FLS cytokines expression profile, we first performed an ELISPOT of 105 cytokines and proteases, comparing activated and non‐activated cell supernatants from HA and non‐HA‐FLS (Figure 1). The Human XL Cytokine Array detects multiple cytokines, chemokines, growth factors and other soluble proteins in cell culture supernates. Concerning all of the cytokines and protease analysed, we demonstrated significant variations essentially in inflammatory process compared to other functions. Between non‐HA‐FLS and HA‐FLS under steady‐state condition (without LPS activation [Medium]), particularly in the expression of Macrophage migration inhibitory factor (MIF), dipeptidyl peptidase IV (DDP‐IV) and Emmprin (Involved in immune intercellular recognition), heavily involved in the control of inflammation (Table 1). As TLR4 lead to a strong activation of NF‐κB pathways, we proposed to use a ligand of TLR4 as lipopolysaccharide (LPS). Between non‐HA‐FLS and HA‐FLS after LPS activation from *Salmonella abortus equi* (1 µg/mL), we found significant variations in 13 expressed cytokines, mostly involved in the control of innate immunity and macrophage maturation (IL‐1α, MCP‐1, M‐CSF, MIF and MIP‐3α), but also in adaptive immunity process (IFN‐γ, IL‐5 and IL‐22), angiogenesis (Thrombospondin 1, Pentraxin 3), bone remodelling (osteopontin and vitamin D BP) and iron metabolism (TfR) (Table 1).

**TABLE 1:** Differences of cytokines in Cytokine Array expression –HA‐FLS versus Non‐HA‐FLS

		Medium	LPS
Control of innate immunity and macrophage maturation	IL‐1α	=	↓↓
	IL‐6	=	=
	IL‐8	=	=
	MCP‐1	=	↑↑
	M‐CSF	=	↓↓
	MIF	↓↓	↓
	MIP‐3α	=	↑↑
Adaptive immunity process	IFN‐γ	=	↓↓
	IL‐5	=	↓↓
	IL‐17A	=	=
	IL‐22	=	↑↑
Angiogenesis	Pentraxin 3	=	↓↓
	Thrombospondin 1	=	↑↑
Bone remodelling	Osteopontin	=	↓↓
	Vitamin D BP	=	↓↓
Iron metabolism	TfR	=	↓↓
Others	DPPIV	↓	↑↑
	Emmprin	↓↓	=
	ENA‐78	=	=
	FGF‐basic	=	=
	FGF‐19	=	=
	GRO α	=	=
	Serpin E1	↓	=

Note

Some significant variations have been demonstrated between HA‐FLS (5 patients) and non‐HA‐FLS (3 patients) with LPS (1 µg/mL) or without LPS. These variations could be classified into different groups: innate immunity and macrophage maturation, adaptive immunity process, angiogenesis, bone remodelling, iron metabolism and others. =: 0 (no difference); ↓↓: 0 ‐ 0.5 (decrease in cytokine expression between 0% and 50%); ↓: 0.5 ‐ 0.75 (decrease in cytokine expression between 50% and 75%); ↑: 1 ‐ 1.5 (increase in cytokine expression between 100% et 150%); ↑↑: 1.5 ‐ 2 (increase in cytokine expression between 150% et 200%). HA‐FLS, FLS of haemophilic patients; non‐HA‐FLS, FLS of non‐haemophilic patients; LPS, lipopolysaccharide; DPPIV, dipeptidyl peptidase; ENA, epithelial neutrophil‐activating protein; FGF, fibroblast growth factor; GRO, growth‐regulated oncogene; IFN, interferon; IL, interleukin; MCP, monocyte chemoattractant protein; M‐CSF, monocyte colony‐stimulating factor; MIF, macrophage migration inhibitory factor.

**FIGURE 2:**
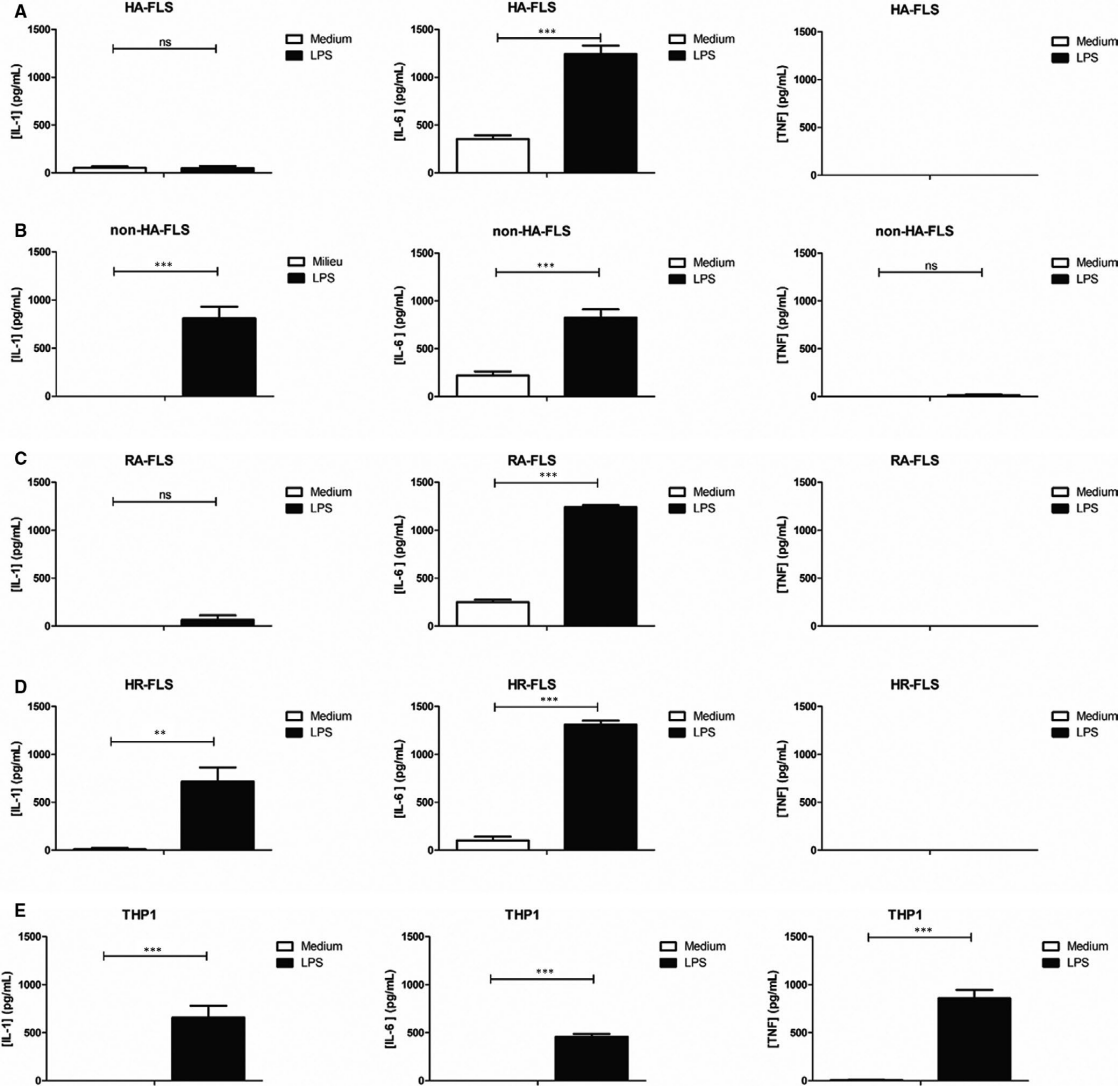
LPS induced a unique release of inflammatory cytokines in HA‐FLS, with in particular a lack of secretion of IL‐1α and TNF‐α. Effect of LPS of IL‐1α, IL‐6 and TNF‐α release by HA‐FLS (A), non‐HA‐FLS (B), RA‐FLS (C), HR‐FLS (D) and THP‐1 cells (E). Cells are activated with LPS (1 µg/mL) for 6h; cytokines releases (IL‐1α, IL‐6 and TNF‐α) were determined by ELISA in culture supernatants. Data are expressed as the mean of triplicate samples ± SD and are representative of three independent experiments (***P < .0001, **P < .001, ns = not significant). LPS: lipopolysaccharide; HA‐FLS: FLS of haemophilic patients; non‐HA‐FLS: FLS of non‐haemophilic patients; RA‐FLS: FLS of rheumatoid arthritis (commercial cells); HR‐FLS: normal FLS (commercial cells); THP‐1: human monocytes; IL‐1α: interleukin‐1; IL‐6: interleukin‐6; TNF‐α: tumour necrosis factor

Given that innate immunity cytokines appeared particularly dysregulated, in an effort to confirm the inflammatory signature of HA‐FLS, we focused on the cytokine expression of three proinflammatory proteins (IL‐1α, IL‐6 and TNF‐α) after 6 hours of activation with LPS. These three cytokines were selected because they are widely involved in the pathophysiology of several arthritis including rheumatoid arthritis; pathology in which IL‐1, IL‐6 and TNF‐α are therapeutic targets. Activated cell supernatants were analysed by means of ELISA. Although LPS stimulation induced IL‐6 secretion in all cell cultures (Figure 2), activated HA‐FLS (Figure 2A) did not release any detectable amount of IL‐1α in cell culture supernatants in a similar manner than RA‐FLS (Figure 2C), whereas non‐HA‐FLS (Figure 2B), HR‐FLS (Figure 2D) and THP‐1 cell lines (Figure 2E) did release significant detectable amounts of IL‐1α. As previously described, no TNF‐α expression was found after LPS stimulation in any of the fibroblast subtypes, as compared to the THP‐1 cell line (Figure 2E).^15^

### 3.3 IL‐1α inflammatory cytokine synthesis is repressed post‐transcriptionally by a miRNA‐dependent pathway in LPS‐activated HA‐FLS

**FIGURE 3:**
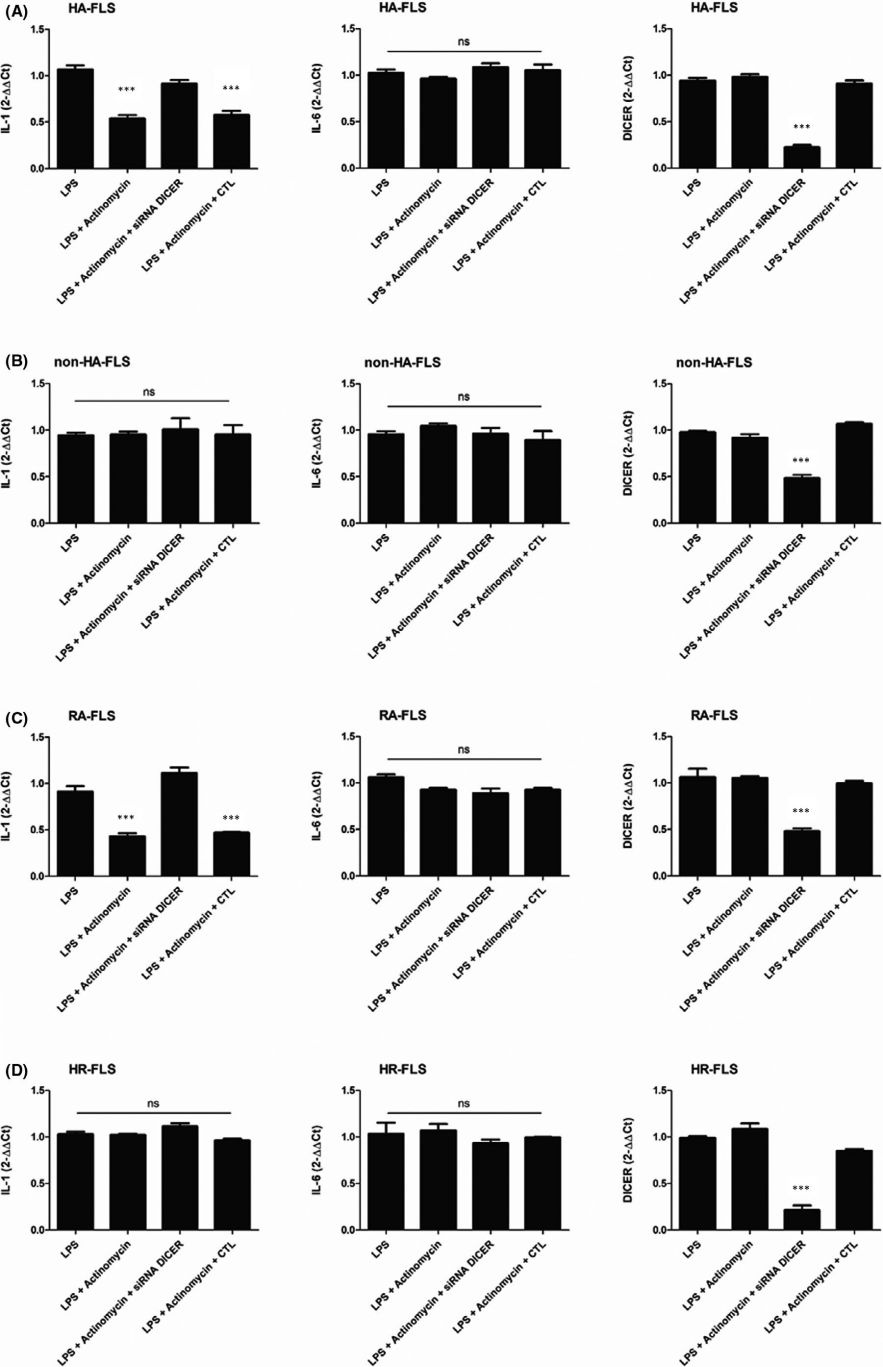
Inflammatory cytokine synthesis is repressed post‐transcriptionally by a miRNA‐dependant pathway in LPS‐activated HA‐FLS. Effect of LPS on IL‐1α, IL‐6 and DICER mRNA expression by HA‐FLS (A), non‐HA‐FLS (B), RA‐FLS (C) and HR‐FLS (D); transfected or not transfected with siRNA DICER. Cells were transfected with siRNA DICER or with the HiPerFect Transfection negative control (CTL). LPS (1 µg/mL) activation of transfected cells was performed 24 h post‐transfection for 6 h and incubated for another 2 h with actinomycin D (5 µg/mL). Non‐transfected HA‐FLS, non‐HA‐FLS, RA‐FLS and HR‐FLS were activated with LPS (1 µg/mL) for 6 h and incubated for another 2 h with actinomycine D (5 µg/mL). mRNA levels were determined using quantitative RT‐PCR. Results were normalized to ACTIN and GAPDH and expressed as the 2‐ΔΔ Ct expression compared with samples from cells incubated in LPS. Data are expressed as the mean of triplicate samples ± SD and are representative of three independent experiments (***P < .0001, ns = not significant). LPS: lipopolysaccharide; HA‐FLS: FLS of haemophilic patients; non‐HA‐FLS: FLS of non‐haemophilic patients; RA‐FLS: FLS of rheumatoid arthritis (commercial cells); HR‐FLS: normal FLS (commercial cells); THP‐1: human monocytes; IL‐1α: interleukin‐1; IL‐6: interleukin‐6; mRNA: RNA messenger

To determine the mechanism underlying the regulation of inflammatory cytokine expression in activated HA‐FLS, we investigated the effect of LPS treatment on IL‐1α and IL‐6 mRNA accumulation in HA, non‐HA, RA, as well as HR‐FLS. RT‐qPCR was performed with RNA isolated from FLS that either had or had not been activated with 1µg/mL LPS for 6 hours. LPS treatment of HA‐FLS (Figure 3A), non‐HA‐FLS (Figure 3B), RA‐FLS (Figure 3C) and HR‐FLS (Figure 3D) resulted in a detectable accumulation of either IL‐1α or IL‐6 mRNA within 6 hours. A similar expression of each mRNA was found between HA and non‐HA‐FLS.

To investigate the mechanism responsible for the lack of IL‐1α protein synthesis in LPS‐activated HA‐FLS and RA‐FLS, as compared to non‐HA‐FLS and HR‐FLS, cells were incubated with LPS for 6 hours, and then for another 2 hours, with 5 µg/mL actinomycin D. After 2 hours of actinomycin D treatment, IL‐1α mRNA decreased drastically in both LPS‐activated HA‐FLS (Figure 3A) and LPS‐activated RA‐FLS (Figure 3C), yet neither in LPS‐activated non‐HA‐FLS (Figure 3B) nor HR‐FLS (Figure 3D). The expression of IL‐6 mRNA was not significantly impacted after actinomycin D treatment in any activated FLS. These results indicate that de novo synthesized IL‐1α mRNA is particularly unstable in both LPS‐activated HA‐FLS and RA‐FLS.

Therefore, to demonstrate the role of miRNAs in the mRNA instability of HA‐FLS, we proceeded to transfect FLS with siRNA targeting human DICER (siRNA‐DICER). After 24 hours of siRNA‐DICER or negative control transfection at 50 nM, FLS were first activated with LPS for 6 hours and then for another 2 hours with actinomycin D. The efficacy of DICER silencing was confirmed using RT‐qPCR of DICER mRNA. After siRNA‐DICER transfection into HA‐FLS (Figure 3A), non‐HA‐FLS (Figure 3B), RA‐FLS (Figure 3C) and HR‐FLS (Figure 3D), DICER mRNA expression was drastically down‐regulated compared to control transfection. Moreover, following actinomycin D treatment, siRNA‐DICER transfection restored IL‐1α mRNA expression in LPS‐induced FLS in both HA‐FLS (Figure 3A) and RA‐FLS (Figure 3C).

### 3.4 Microarray analysis of miRNA profile of HA‐FLS shows a single miRNA signature close to that of inflammatory arthritis and diseases

As miRNAs was likely involved in the post‐transcriptional control of inflammatory cytokines in HA‐FLS, we analysed the global expression profile of miRNAs using a microarray‐based approach after exposing HA and non‐HA‐FLS to LPS or medium in an effort to identify miRNAs. FLS were stimulated with LPS (1 µg/mL) or medium for 6 hours, with the extracted RNAs compared using a DNA microarray containing 2578 probes complementary to miRNAs of human origin. HA‐FLS were collected from patients with haemophilia A, haemophilia B and Type 3 von Willebrand disease to be independent of the potential impact of the constitutive genetic mutation. We also extracted RNA from FLS at three different passages so that any variation in miRNA expression levels would not be influenced by culture conditions.

According to the miRNA microarray results from the FLS, we found that more than a thousand miRNAs were differentially expressed in all HA‐FLS and non‐HA‐FLS (P50, p0.05, f1.2) (Figure S2). We then focused on differences in miRNA expression levels between HA‐FLS and non‐HA‐FLS. As a result, 14 miRNAs in the steady‐state condition with medium control (Table 2) were revealed to display significantly different expression levels, as did 16 different miRNAs after LPS stimulation (Table 3). Most of these 30 miRNAs could potentially target mRNAs involved in inflammatory processes directly or indirectly, such as proinflammatory cytokines (IL‐1α and L‐6 with miR‐146 or miR‐224, TNF‐α with miR‐146a‐5p or miR185a‐5p or miR125b‐2‐3p, etc) or specific signalling pathways (PI3K and micro‐RNA‐204‐3p, SOCS3/STAT3 and miR‐196b‐5p, etc).

**TABLE 2:** Microarray analysis of miRNA profile of HA‐FLS shows a single miRNA signature close to that of inflammatory arthritis and diseases

miRNA	Ratio
hsa‐miR‐181b‐5p	0.5792666
hsa‐miR‐10b‐5p	0.6143269
hsa‐miR‐214	0.8012025
hsa‐miR‐4801	0.8105773
hsa‐miR‐3942‐5p	1.3223936
hsa‐miR‐106b‐3p	1.380158
hsa‐miR‐1271‐5p	1.6083829
hsa‐miR‐25‐3p	1.6462257
hsa‐miR‐331‐3p	1.6734163
hsa‐miR‐4284	1.7212519
hsa‐miR‐221‐5p	1.7217574
hsa‐miR‐154‐5p	1.8831481
hsa‐miR‐146b‐5p	1.9955731
hsa‐miR‐196b‐5p	2.4280967

Note

The global expression profile of miRNAs is analysed using a microarray‐based approach after exposing HA and non‐HA‐FLS to LPS. FLS were stimulated with medium (Table 2) and LPS (Table 3) for 6 hours, and the extracted RNAs were compared using a DNA microarray containing 2578 probes complementary to miRNAs of human origin. A HA‐FLS/non‐HA‐FLS miRNA ratio was used to determinate expression levels of each miRNA. After used of a significant cut‐off ratio, respectively, at < 0.75 and > 1.5, 14 miRNAs had significantly different expression levels after medium stimulation (Table 2) and 16 miRNAs had significantly different expression levels after LPS stimulation (Table 3).

**TABLE 3:** Microarray analysis of miRNA profile of HA‐FLS shows a single miRNA signature close to that of inflammatory arthritis and diseases

miRNA	Ratio
hsa‐miR‐146a‐5p	0.2782165
hsa‐miR‐6511b‐5p	0.4940837
hsa‐miR‐1246	0.5300267
hsa‐miR‐204‐3p	0.6569294
hsa‐miR‐4734	1.2227555
hsa‐miR‐212‐5p	1.3988092
hsa‐miR‐559	1.4678181
hsa‐miR‐1180‐3p	1.5138389
hsa‐miR‐125b‐2‐3p	1.5144118
hsa‐miR‐548u	1.5837457
hsa‐miR‐299‐3p	1.5844876
hsa‐miR‐1298‐3p	1.6381411
hsa‐miR‐130b‐3p	1.6484885
hsa‐miR‐1271‐5p	1.991351
hsa‐miR‐585‐5p	2.1910996
hsa‐miR‐34a‐3p	2.314242

Note

The global expression profile of miRNAs is analysed using a microarray‐based approach after exposing HA and non‐HA‐FLS to LPS. FLS were stimulated with medium (Table 2) and LPS (Table 3) for 6 hours, and the extracted RNAs were compared using a DNA microarray containing 2578 probes complementary to miRNAs of human origin. A HA‐FLS/non‐HA‐FLS miRNA ratio was used to determinate expression levels of each miRNA. After used of a significant cut‐off ratio, respectively, at < 0.75 and >1.5, 14 miRNAs had significantly different expression levels after medium stimulation (Table 2) and 16 miRNAs had significantly different expression levels after LPS stimulation (Table 3).

Interestingly, some of these miRNAs, such as miR‐146‐5p,^16^, ^17^ miR‐204‐3p,^18^ miR‐130b‐3p,^19^ miR‐25‐3p,^20^ miR‐10b‐5p,^21^ miR‐221‐5p,^22^ miR‐4284^23^ and miR‐3942‐5p^24^, have previously been described as being involved in regulating inflammation in RA, Sjögren's syndrome, lupus, familial Mediterranean fever, ankylosing spondylitis and Crohn's disease. Others have been described as playing a major role in inflammatory control, such as miR‐1246^25^, ^26^ in macrophage polarization, miR‐585‐5p^27^ in oxidative stress through PARP1, miR‐34‐3p^28^, ^29^ in fibroblast proliferation and TNF‐α expression, miR‐196b‐5p^30^ in SOCS3/STAT3 activation, miR‐146b‐5p in IL‐1α/IL‐6/IL‐8 control through the TLR4 pathway^31^, ^32^ and miR‐224^33^ through the PTEN pathway. Many of these have been reported to play a role the pathogenesis of solid and haematologic tumours. However, some of these miRNAs have not yet been described in any disease or process, such as miR‐6511b‐5p and miR‐4801.

### 3.5 Correlation between acquired miRNA profile in HA‐FLS and repeated plasma exposure

To demonstrate that epigenetic changes are due to a chronic contact of blood with FLS, we propose to use an in vitro model of onset haemophilic arthropathy after repeated contact between plasma and normal HR‐FLS.

**FIGURE 4:**
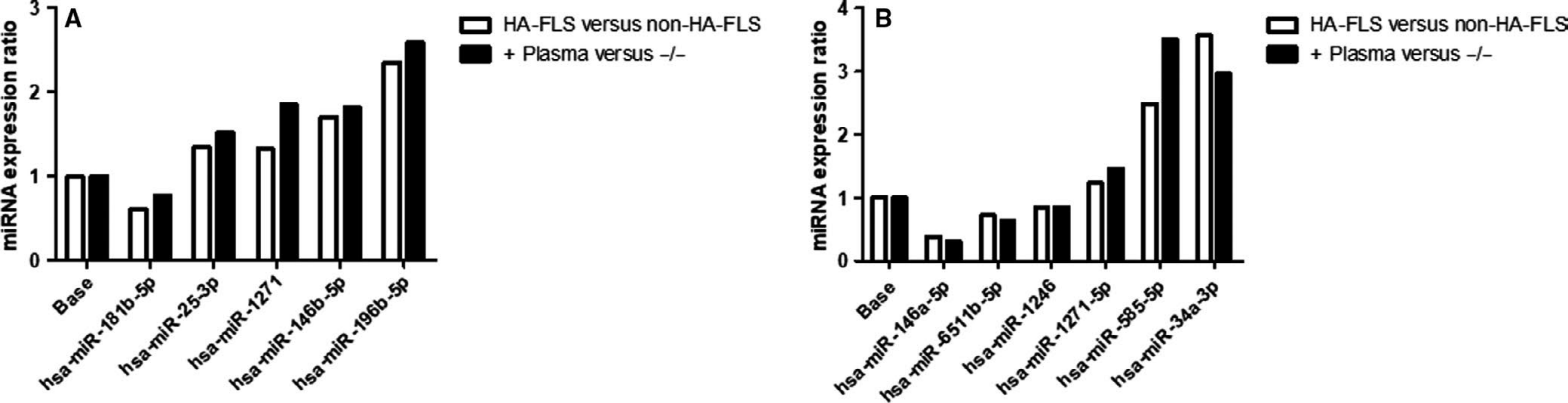
Chronic plasma exposure in normal HR‐FLS lead to the same miRNA profile than HA‐FLS. qRT‐PCR was performed of 11 selected miRNAs from microarray discovery in HA and non‐HA‐FLS and also in plasma exposed normal HR‐FLS (+ plasma) and non‐exposed HR‐FLS (‐/‐), on the condition medium (A) (has‐miR‐181b‐5p, hsa‐miR‐25‐3p, has‐miR‐1271‐5p, hsa‐miR‐146b‐5p and hsa‐miR‐196b‐5p) and on the condition LPS (B) (hsa‐miR146a‐5p, hsa‐miR‐6511b‐5p, hsa‐miR1246, hsa‐miR‐1271‐5p, hsa‐miR‐585‐5p and hsa‐miR34a‐3p). miRNA expression was verified using miScript SYBER Green Kit and miScript Primer Assays. The 2‐ΔΔCt method was used to calculate relative changes in miRNA expression. Results were normalized to SNORD and RNU. Data are expressed as the mean of triplicate samples ± SD and are representative of three independent experiments (***P < .0001, **P < .001, *P < .01)

We first selected 11 miRNAs mostly impacted on the miRNome, respectively, 6 miRNAs in LPS condition and 5 miRNAs in steady‐state condition. MiR RT‐qPCR was initially carried out in HA and non‐HA‐FLS, confirming the same variations as observed in the MiRNome analyse, in the expression ratio of these 11 miRNAs in FLS activated by either medium (Figure 4A) or LPS (Figure 4B).

We also compared expression of these 11 miRNA after 3 weeks of repeated exposure of normal HR‐FLS by plasma or medium. We observed the same variations between plasma exposed versus non‐exposed HR‐FLS that HA versus non‐HA‐FLS, after medium activation (Figure 4A) or LPS (Figure 4B).

### 3.6 Transcriptomic network potentially impacted by miRNA expression in HA‐FLS: From inflammatory to ubiquitous changes

To further investigate the potential impact of miRNA epigenetic changes in FLS following chronic interaction with blood, we performed a computer analysis pertaining to the targets of previous 11 miRNAs selected. As each miRNA is likely to possess several hundred mRNA targets, we focused on mRNAs potentially interacting with at least three of the 11 selected miRNAs. The extracted RNAs were compared using a DNA microarray containing 2578 probes complementary to miRNAs of human origin.

**FIGURE 5:**
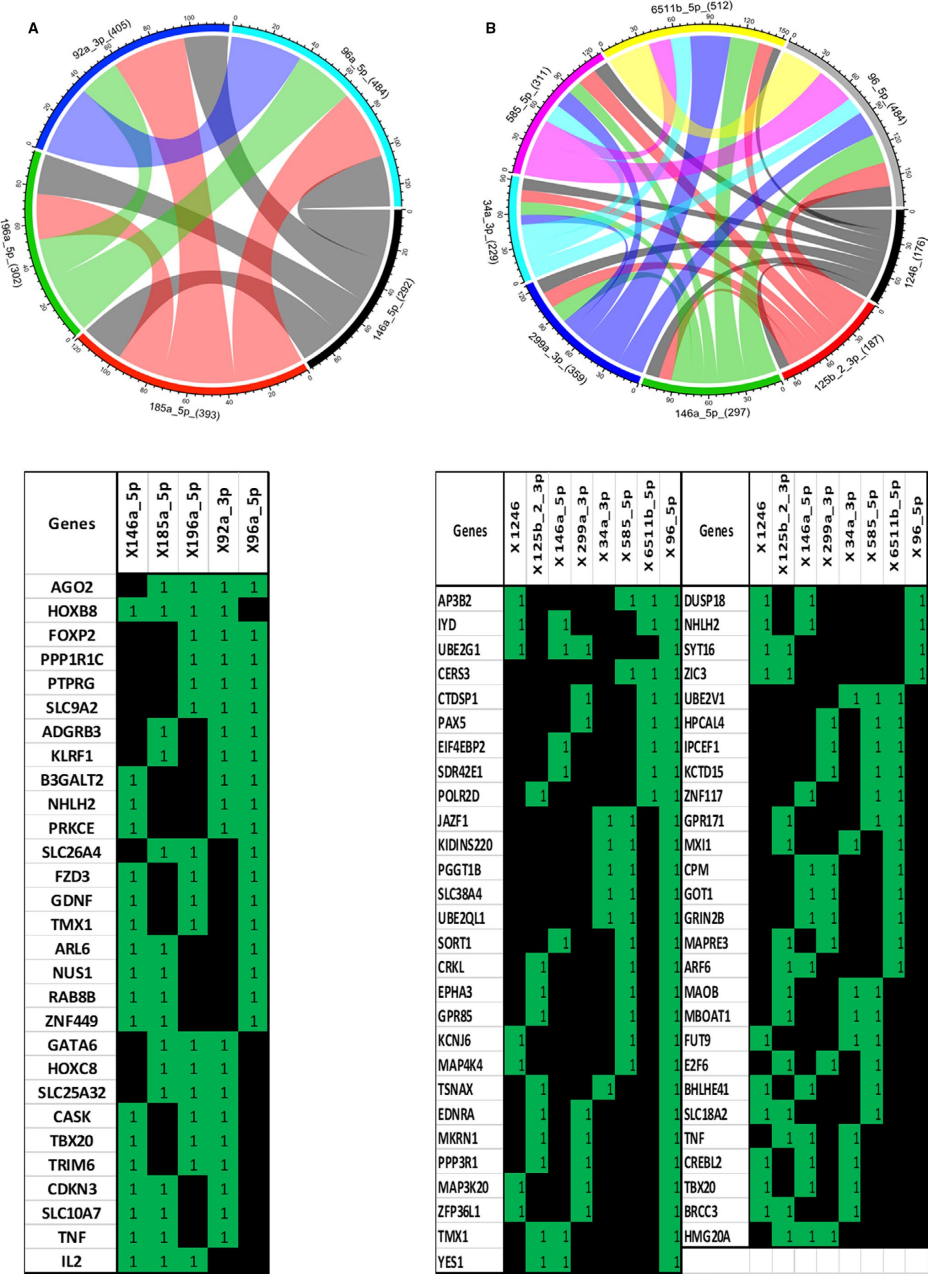
Transcriptomic network potentially impacted by miRNA expression in HA‐FLS: from inflammatory to ubiquitous changes. The extracted RNAs were compared using a DNA microarray containing 2578 probes complementary to miRNAs of human origin. Chord diagram of the regulated genes (A: Medium; B: LPS) shared between the miRs. Each chord represent a number genes regulated by two miRs. The total number of regulated genes by each miR is mentioned between brackets. Table of genes (A: Medium; B: LPS) targeted by at least 3 miRs (according to Ingenuity IPA), 1/green if a gene is a target of a miR and 0/black if not

Under steady‐state condition without LPS activation, we found that 29 mRNAs could be targeted (Figure 5A). Protein encoded by these mRNAs is known to be highly involved in controlling gene transcription (FOXP2, NHLH2, etc) and post‐transcriptional regulation (AGO2), and other to transmembrane protein control (B3GALT2, PRKCE, etc) or metabolism changes (PPP1R1C, PTPRG, etc). However, some proteins encoded by miRNAs are known to play a specific role in osteocartilaginous changes and bone remodelling (FZD3, HOXC8 and SLC10A7), and others are involved in inflammatory process (ADGRB3, KLRF1, GDNF, IL2 and TNF‐α).

Upon LPS activation, we detected that 55 mRNAs could be targeted by three or more selected miRNAs (Figure 5B). As in steady‐state condition, proteins encoded by these mRNAs are known to be particularly involved in protein trafficking, processing and destruction, as well as inflammatory pathways. These proteins eoncoded by mRNAs are highly involved in controlling gene transcription and post‐transcriptional regulation, while playing a specific role in angiogenesis and osteocartilaginous changes.

## 4 DISCUSSION

In this study, we demonstrate that episodic blood interaction is likely to induce durable epigenetic changes in specific resident cells that do not normally interact directly with blood. In haemophilia, hemarthrosis is the most common complication affecting haemophilic patients, and repeated intra‐articular bleeds lead to progressive joint destruction.^34^ The resident joint cells, namely the FLS, are repeatedly exposed to blood during flare‐ups of hemarthrosis. HA is a unique form of arthritis characterized by a repeated interaction between blood and FLS. Currently, there are two theories that are not exclusive, as for the nature and type of this arthritis. On the one hand, HA may result from a mechanical process much like osteoarthritis with little involvement of inflammation. On the other hand, HA may be envisioned as an inflammatory process underlying HA similar to that of RA.^35^, ^36^, ^37^ Our results provide evidence for the second hypothesis.

Given that so far little data on the use of primary FLS from haemophilic patients are available, we have first characterized FLS and shown that they were entirely composed of synoviocytes. The results of flow cytometry confirmed that the primary FLS exhibited exactly the same profile than the FLS cell line, with no marker of cells of other origin found, in particular no macrophages (Figure S1). Therefore, the cytokines, mRNAs and miRNA epigenetic changes observed solely involved FLS.

Cytokine expression profile using ELISPOT analysis between HA‐FLS and non‐HA‐FLS, either activated or not by LPS, seems to be in line with the inflammatory theory (Figure 1 and Table 1). Cytokines involved in innate immunity were particularly impacted by the expression difference between non‐HA and HA‐FLS. We also noticed that HA‐FLS were likely to participate in the bone remodelling process and iron recycling, which confirms the central role of FLS in the control of haemophilic arthropathy physiopathology. We then observed that HA‐FLS and RA‐FLS did not secrete IL‐1α after LPS treatment, whereas in non‐HA‐FLS, THP‐1 cell lines and HR‐FLS IL‐1α were detected in supernatants (Figure 2). The reasons for this paradoxical down‐regulation of IL‐1α in LPS‐induced HA‐FLS and RA‐FLS are unknown, whereas IL‐1β’s role in the pathogenesis of HA has been clearly demonstrated.^38^ Indeed, when activated by erythrocyte phagocytosis, monocytes and macrophages were reported to produce IL‐1β and TNF‐α in the joint after hemarthrosis, which could explain the control of FLS expression by a negative feedback pathway of IL‐1α but this hypothesis needs to be confirmed. Following LPS activation of FLS in RA, non‐expression of TNF‐α has been reported due to epigenetic regulation.^17^ Of note is that TNF‐α is likewise key to inflammation in HA, as recently published with regard to the role of the iRhom2/ADAM17/TNF‐α pathway^39^ and also as a crucial mediator of proliferative synovitis in haemophilia A.^39^

In contrast to TNF‐α and IL‐1α, we did observe high IL‐6 expression levels after LPS treatment in HA‐FLS, in line with previous data that demonstrated the utility of targeting IL‐6 in HA.^41^ Interestingly, the cytokine profile of LPS‐induced HA‐FLS mostly correlated with the potential post‐transcriptomic control of the 30 miRNAs selected (Figure 1 and Table 3). After LPS activation, we observed that HA‐FLS up‐regulated the expression of cytokines involved in macrophage polarization (also control by miR‐1246) and modulated the TH17 pathway (miR‐10b‐5p) involved in ankylosing spondylitis. Moreover, most of the other miRNAs may influence inflammatory cytokine expression directly or indirectly, for instance through the NFkB, PI3K or JAK/STAT pathways (miR‐146 and miR‐196b‐5p, the miRNAs with the highest expression ratios).

We confirm that the FLS cytokine profile was influenced by mRNA instability, especially in HA‐FLS after actinomycin D treatment (Figure 3). However, there is more than one process involved in mRNA stability, particularly the 5’ and 3’ UTR mRNA regulation region with RNA‐binding proteins or miRNA complexes. To demonstrate that miRNAs were primarily involved in mRNA instability in HA‐FLS, we decided to silence DICER, an essential enzymatic step for miRNA maturation. Following siRNA DICER transfection, LPS‐induced HA‐FLS did not exhibit proinflammatory mRNA instability after actinomycin D treatment (Figure 3A). The transcriptomic network of the selected miRNAs in LPS‐induced HA‐FLS revealed the inflammatory process and protein synthesis to be severely impacted (Figure 5B and Table 3). Moreover, on analysing the transcriptomic network of the selected miRNAs in steady‐state HA‐FLS without LPS activation, we observed that pathways involved in angiogenesis, osteocartilaginous changes and inflammatory control process were impacted without any stimulation (Table 2 and Figure 5A).

To confirm that repeated hemarthrosis could induced those miRNA epigenetic changes into articular resident cells, we proposed to create an in vitro model of haemophilic arthropathy with normal HR‐FLS repeatedly exposed to plasma every 3 days for 3 weeks. We observed that selected miRNAs acquired the same expression patterns as the HA‐FLS, in medium and LPS conditions (Figure 4A et 4B). It would also be interesting to determine the ‘plasma free time’ needed for the plasma exposed HR‐FLS to restore the same miRNA profile than non‐exposed. However, FLS culture conditions and passages induce major limiting factors to perform this experiment. Indeed, in this model, we used normal plasma samples with the same volume and duration of exposure. In a clinical point of view, when a haemophilic patient present intra‐articular bleeding, blood in contact with the synovial membrane could have a variable volume and duration of exposure, with the presence of blood cells and different concentration of proteins especially coagulation factors. These elements could also modulate FLS signature after blood exposure, and this could be an explanation of variability of joint damage and degree of haemophilic arthropathy between each patient.

These findings confirm the clinical theory that just a few repeated episodes of hemarthrosis are sufficient to cause irreversible changes in the joint, while triggering the process of progressive articular degradation. Targeting the goal of ‘very few or no bleeds’ in haemophilic patients is therefore justified, primarily designed to avoid these irreversible epigenetic changes in synoviocytes. Recently, some data demonstrated that other triggers than classical FVIII or FIX concentrates could protect haemophilic patients from joint bleeding, such as thrombin‐activatable fibrinolysis inhibitors or TAFI, which underscores the relevance of clot protection in addition to clot formation.^42^ Different prohemostatic strategies must thus be considered in an effort to avoid definite haemophilia joint bleeding.

It could be interesting to extend this concept of interaction between blood and cells to other cell types, which, under normal conditions, do not interact with blood, such as neuronal and microglial cells following repeated intracranial bleeds in certain sports (boxing, American football, etc).

In conclusion, these findings demonstrate that resident cells in the joints of haemophilic patients develop a unique proinflammatory pattern, close to rheumatoid arthritis, due to durable miRNA changes owing to repeated episodes of hemarthrosis.

## CONFLICT OF INTEREST

None of the authors have any conflicts of interest to disclose, relating to this study.

## AUTHORS’ CONTRIBUTION

Sandra Mignot: Conceptualization (equal); Formal analysis (equal); Resources (equal); Validation (equal); Visualization (equal); Writing‐original draft (equal); Writing‐review & editing (equal). Nicolas Cagnard: Formal analysis (supporting); Methodology (equal); Writing‐original draft (supporting). Benoit Albaud: Formal analysis (supporting); Methodology (equal); Writing‐original draft (supporting). Cecile Bally: Conceptualization (supporting); Methodology (supporting); Visualization (supporting); Writing‐original draft (equal). Justine Siavellis: Investigation (supporting). Olivier Hermine: Conceptualization (equal); Formal analysis (equal); Funding acquisition (equal); Investigation (equal); Methodology (equal); Resources (equal); Validation (equal); Writing‐original draft (equal); Writing‐review & editing (equal). Laurent Frenzel: Conceptualization (lead); Formal analysis (equal); Funding acquisition (equal); Investigation (equal); Methodology (equal); Project administration (lead); Resources (equal); Supervision (equal); Validation (lead); Writing‐original draft (lead); Writing‐review & editing (lead).

## Supporting information

Supplementary Material
