# Supplementary material for: Unique inflammatory signature in haemophilic arthropathy: miRNA changes due to interaction between blood and fibroblast‐like synoviocytes
Source: J Cell Mol Med. 2020 Nov 7;24(24):14453–66. doi: 10.1111/jcmm.16068 (PMC7753994; doi:10.1111/jcmm.16068)
Supplement: Supplementary file 1 — Supplementary Material [file JCMM-24-14453-s001.docx]

**MATERIALS AND METHODS (Supplementary data)**

Reagents

Cell culture media (RPMI 1640, M199), fetal bovine serum, L-glutamine, penicillin, streptomycin, amphotericin B, and trypsin reagent were purchased from Thermo Fisher Scientific. Lipopolysaccharides (LPS) were obtained from Salmonella abortus equi, Type XI collagenase and actinomycin D from Streptomyces sp. from Sigma-Aldrich. The Human Cytokine Array Kit and enzyme immunoassay kit for detecting human IL-1α, IL-6, and TNF-α were obtained from R&D Systems. The iScript Reverse Transcription Supermix for the real-time quantitative polymerase chain reaction (RT-qPCR), and SsoFast EvaGreen Supermix, were obtained from Bio-Rad. The RNeasy Plus Mini Kit and miScript System were obtained from Qiagen (Courtaboeuf, France). Flow cytometry antibodies and corresponding isotypes were obtained from Becton Dickinson (LePontdeClaix, France).

Cell activation

FLS (5.10^5^cells) and THP-1 (10^7^cells) were stimulated with 1 mL of either medium alone or medium containing LPS (1 µg/mL) for 6 hours. After stimulation, supernatants were harvested and assayed for cytokine content using commercially available ELISA tests.

Flow cytometry

For the expression of the cluster of differentiation CD45, CD55, CD68, CD90, and CD106 in human FLS, HFLS cell lines (RA-HFLS, HR-FLS) and THP-1 cells were measured using a flow cytometry assay^15^. After culture, the cells were washed twice with phosphate buffered saline (PBS), then incubated for 20 minutes at room temperature in the dark with a mixture of “Krome Orange-conjugated anti-CD45 (BD Pharmingen, clone MφP9), PE-conjugated anti-CD55 (BD Pharmingen, clone IA10), FITC-conjugated anti-CD68 (BD Pharmingen, clone Y1/82A), BV421-conjugated anti-CD90 (BD Horizon, clone 5B10), and APC-conjugated anti-CD106 (BD Pharmingen, clone 51-10C9)” . Then, after two washes with PBS, the fluorescent cells were analyzed on a FACScan flow cytometer (Gallios, Beckman Coulter) using Kaluza Analysis (Beckman Coulter).

Protein profiling from supernatant

Protein profiling was performed based on the supernatant of cell activation. Some 300 µL of supernatant were loaded on proteome profiler antibody array membranes (Human XL Cytokine Array Kit, R&D Systems), as suggested by the supplier. These membranes were washed and incubated with biotinylated detection antibody cocktail, streptavidin/horseradish peroxidase, and chemiluminescent detection reagents, as suggested by the supplier. Membranes were exposed to a ChemiDoc and analyzed using ImageJ Software.

Image analysis was performed with background subtraction and normalization to membrane reference points. Differences in the expression levels of various proteins were calculated pairwise as fold changes, with comparisons made between HA and non-HA-FLS.

Stimulation of cells for total extraction

Total RNA was extracted from human FLS or THP-1 cells incubated for 6 hours with either medium alone or medium containing LPS used the RNeasy Plus Mini Kit according to the manufacturer’s instructions. Total RNA isolated from the FLS and THP-1 cells was reverse transcribed using the iScript Reverse Transcription Supermix for RT-qPCR, according to the manufacturer’s instructions (Bio-Rad), and amplified.

mRNA decay measurement

The stability of mRNA was assessed by adding 5 µg/mL of actinomycin D to the cell medium following activation with fetal bovine serum to inhibit mRNA transcription. At the indicated time points, it was possible to correlate the relative amount of specific mRNA remaining in each sample with mRNA degradation. Total RNA was extracted 2 hours after treatment with actinomycin D and endogenous mRNA levels were analyzed by RT-qPCR. Because the mRNA levels for GAPDH and β actin were unchanged after actinomycin D treatment, the GAPDH and β actin gene were employed as a reference, and the ratio of IL-1α/IL-6 and GAPDH/β actin in each sample was calculated.

Real-time quantitative polymerase chain reaction

RT-qPCR was performed on a total of 20µL using the SsoFast EvaGreen Supermix (Bio-Rad) and gene specific primers for (i)TNF-α (5’-TCC-TTC-AGA-CAC-CCT-CAA-CC-3’ and 5’-AGG-CCC-CAG-TTT-GAA-TTC-TT-3’), (ii)IL-1α(5’-CCC-ACA-GAC-CTT-CCA-GGA-GAA-T-3’ and 5’-CGA-CAC-CCT-CGT-TAT-CCC-ATG-TGT-CG-3’), (iii)IL-6 (5’-TAC-CCC-CAG-GAG-AAG-ATT-CC-3’ and 5’-TTT-TCT-GCC-AGT-GCC-TCT-TT-3’), (iv)GAPDH (5’-TTG-ATT-TTG-GAG-GGA-TCT-CG-3’ and 5’-GAG-TCA-ACG-GAT-TTG-GTC-GT-3’) and (v) β -actin (5’-CGT-ACC-CAT-CAC-GAT-GCC-AGT-GGT-ACG-3’ and 5’-ACG-TTG-CTA-TCC-AGG-CTG-TGC-3’).

After initial denaturing at 95°C for 3 minutes, the temperatures used were 95°C for 5 seconds, 58°C for 15 seconds, and 72°C for 15 seconds. Some 40 cycles were performed using the C1000 Touch Instrument (Bio-Rad).

RT-qPCR analyses for miRNAs were conducted using the miScript System, and the primers (Qiagen) and RNA concentrations were determined with a NanoDrop instrument (Thermo Fisher). A 1000ng RNA sample was employed for the assays. Reverse transcriptase reactions and RT-qPCR were carried out according to the manufacturer’s protocols. An endogenous control was employed for normalization. All reactions were run in triplicate on a C1000 Touch Instrument (Bio-Rad).

Single interfering RNA transfection

The DICER siRNA used in our study was designed to effectively inhibit DICER activity, being supplied by Qiagen.

Cells were transfected with siRNA using the HiPerFect Transfection Reagent kit (Qiagen). Cells were plated in 24-well plates (1 x 10^3^ cells/well). All assays were performed 24 hours after transfection

Statistical analysis

Statistical analysis was performed using Student’s t-test. Values were compared between different groups. A p-value of <0.05 was considered statistically significant.

**FIGURES (Supplementary data)**


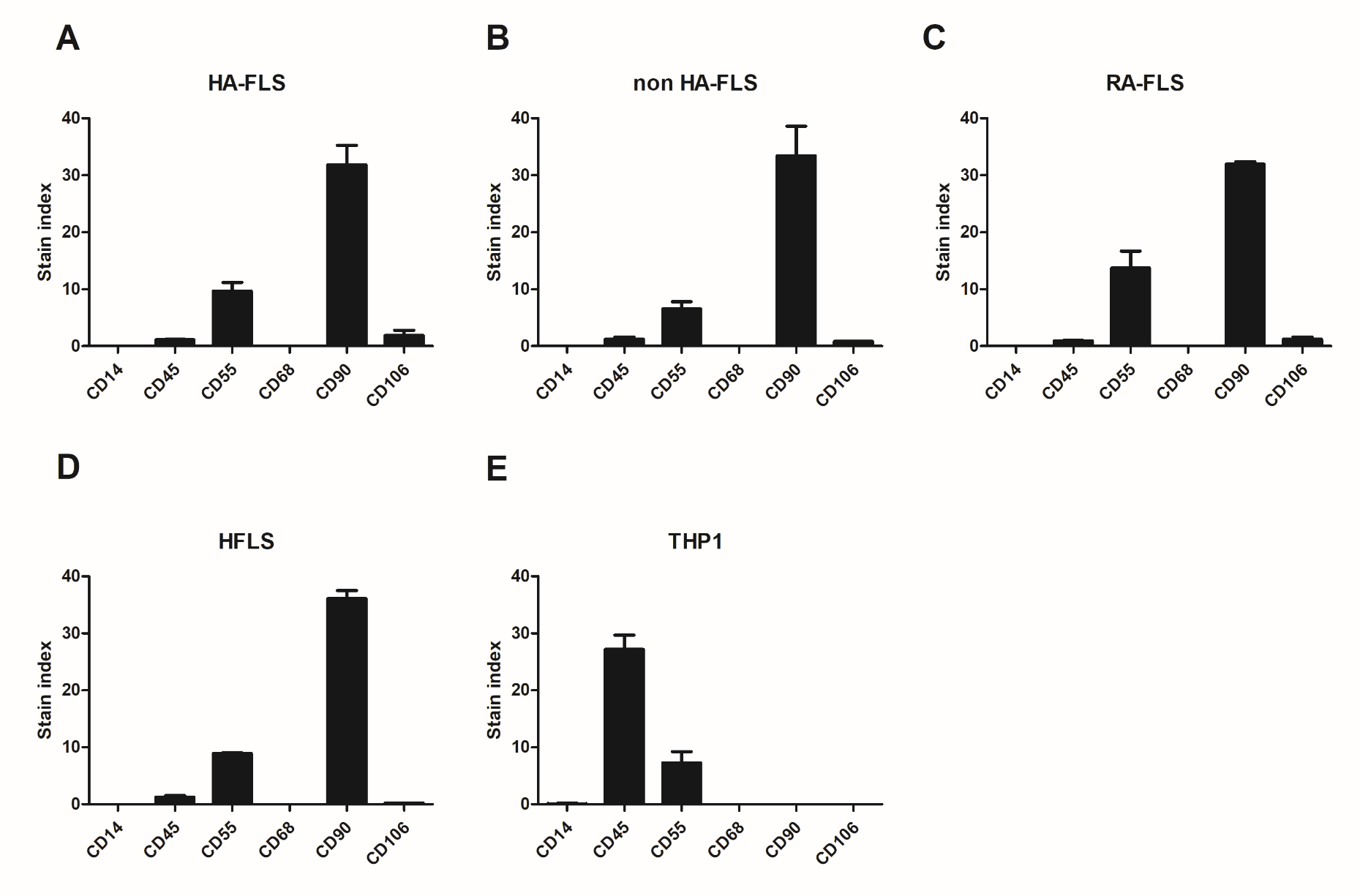


Figure S1 : **Phenotype of different cells : HA-FLS (A), non HA-FLS (B), RA-FLS (C), HR-FLS (D) and THP1 (E).** Expression of CD45, CD55, CD68, CD90 and CD106 markers. HA-FLS (5 patients), non-HA-FLS (3 patients), RA-FLS and HR-FLS are composed of a pure homogenous fibroblastic population and completely differed from the THP-1 cell line. In the joint, we have two specific cells: macrophage-like synoviocytes cells (type A) and fibroblast-like synoviocytes cells. CD68 (lysosomal glycoprotein) is expressed by macrophage-like synoviocytes cells. CD90 (Thy-1) is specific for fibroblast-like synoviocytes and this cells could expressed CD55 (Decay accelerating-factor) and CD106 (Vascular cell adhesion molecule-1) too. CD45 (leukocyte common antigen) is specific for leucocytes cells like THP-1. SI (Stain Index) = (Median of Positive – Median of Negative) / (SD of Negative * 2); HA-FLS : FLS of hemophilics patients ; non-HA-FLS : FLS of non hemophilics patients ; RA-FLS : FLS of rheumatoid arthritis (commercial cells) ; HR-FLS : normal FLS (commercial cells) ; THP-1 : human monocytes. Experiments was performed in triplicate. Statistical analysis was performed using Student’s t-test..


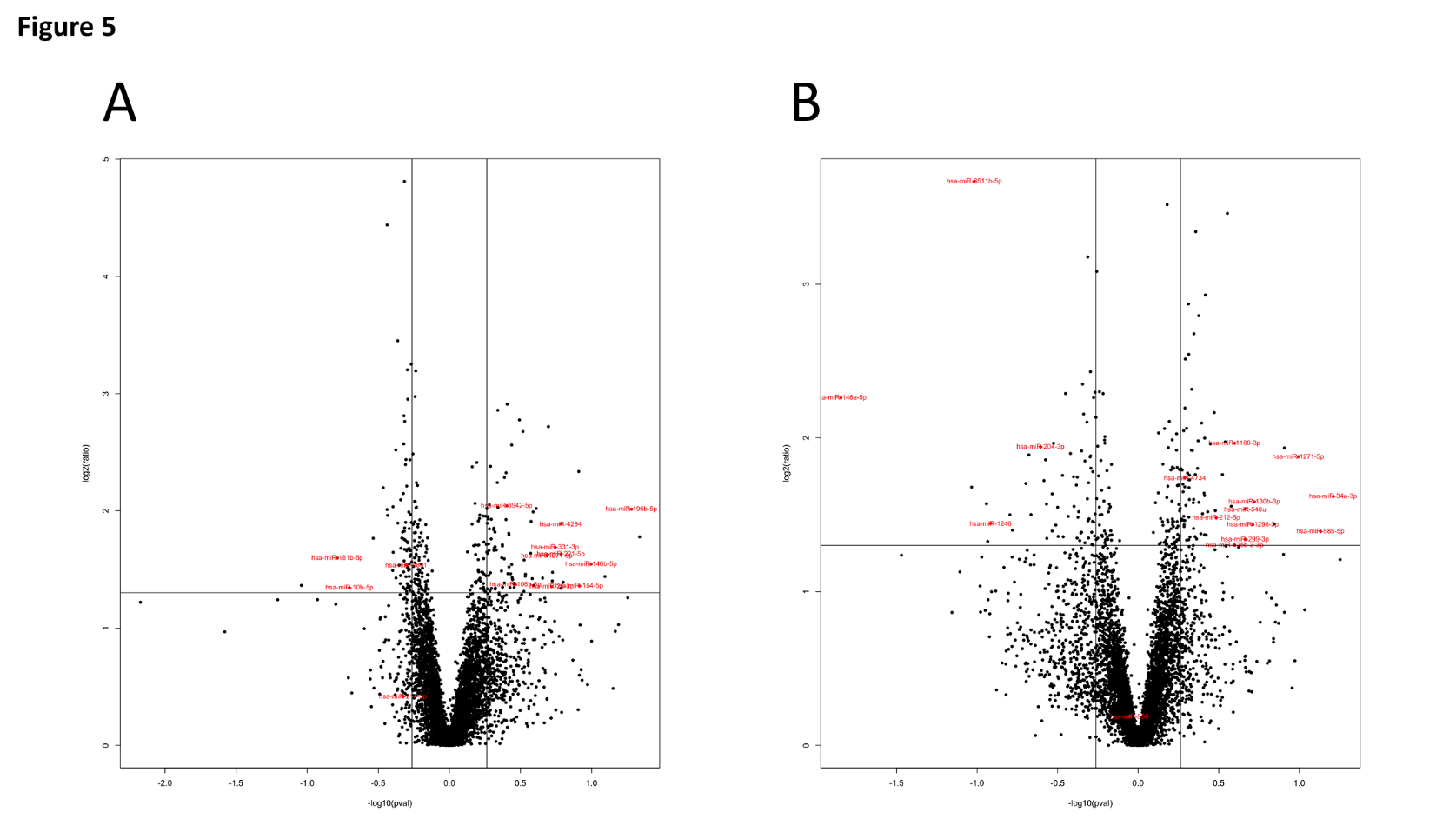


Figure S2 : **Microarray analysis of miRNA profile of HA-FLS versus non HA-FLS.**

HA and non-HA-FLS were stimulated with LPS (1 µg/mL) for 6 hours. The extracted RNAs were compared using a DNA microarray containing 2578 probes complementary to miRNAs of human origin. Volcano plot (A : Medium ; B : LPS) of the differentially expressed genes. Significance (-log10 pvalue) versus log2 fold-change. Genes with statistical significant modulations are over the horizontal bar (pval<=0.05) and genes modulated >= fold 1.2 are outside the vertical bars.
